# Parental involvement could mitigate the effects of physical activity and dietary habits on mental distress in Ghanaian youth

**DOI:** 10.1371/journal.pone.0197551

**Published:** 2018-05-17

**Authors:** Franklin N. Glozah, Kwaku Oppong Asante, Nuworza Kugbey

**Affiliations:** 1 Department of Social and Behavioural Sciences, School of Public Health, University of Ghana, Accra, Ghana; 2 Institute for Psychosocial Research in Child and Adolescent Wellbeing, Accra, Ghana; 3 Department of Psychology, University of Ghana, Accra, Ghana; 4 Department of Family and Community Health, School of Public Health, University of Health and Allied Sciences, Ho, Ghana; Stellenbosch University, SOUTH AFRICA

## Abstract

**Introduction:**

Parental involvement in physical activity and dietary habits have been found to play a substantial role in the mental health of young people. However, there is little evidence about the associations between parental involvement, health behaviours and mental health among Ghanaian youth. This study sought to examine the role of parental involvement in the association between physical activity, dietary habits and mental health among Ghanaian youth.

**Methods:**

Data were obtained from the 2012 Ghana Global School-based Student Health Survey (GSHS). The study population consisted of 1,984 school going youth in high schools with a median age of 15 years old, (53.7%) males. Bivariate and multivariate logistic regression statistical models using complex samples method were performed.

**Results:**

The prevalence of mental distress was 18.1%, 16.6% and 23% for loneliness, feeling worried and suicidal ideation respectively. Younger students were more likely to feel lonely, worried and have suicidal ideation than older students. Students from low socio-economic backgrounds were significantly more likely to report loneliness, worry and suicidal ideation. After adjusting for socio-demographic characteristics, some physical activity and eating habits were associated with experiencing loneliness, worry and suicidal ideation but after introducing parental involvement, there was a decrease in the likelihood of some health behaviour factors in both physical activity and dietary habits to be associated with loneliness, worry and suicidal ideation.

**Conclusion:**

Physical inactivity and poor dietary habits could have a negative effect on mental distress, however, parental involvement could mitigate the impact of these lifestyle habits on mental distress and should therefore be taken into consideration in efforts aimed at encouraging positive lifestyle habits for good mental health among Ghanaian youth.

## Introduction

Mental ill-health is common among young people globally and it has been declared as a chronic diseases of the young [1]. Globally, about 20% of children and adolescents suffer from one form of mental distress or the other [2]. Although there is a paucity of research studies on adolescent mental health in Africa [3], a few studies conducted in Africa found that 1 out of 7 children have significant mental health problems and 1 out of 10 were diagnosed with a mental health problem [4]. Quite recently, about 62% of Ghanaian youth have reported having moderate to high common mental illnesses [5]. This evidence is in consonance with what is already known that mental health problems are a leading cause of disease burden globally including both low-and middle-income economies [6, 7].

Adolescence is a critical period and mental distress during this time in life may impact negatively on academic success [8, 9]. In contrast, when young people have a good sense of mental health they are able to demonstrate good problem-solving skills, social competence and have a sense of purpose which empowers them to recover from adverse circumstances, avoid health impairing habits and live a productive life [7, 10]. Having and/or maintaining good mental health does not come easy as several factors including health impairing behaviours (e.g. physical inactivity and poor dietary habits) impede young people’s ability to good mental health.

Various forms of physical activity are associated with several mental health outcomes in young people. In a review of review study to synthesise evidence on chronic physical activity participation and mental health, Biddle and Asare [11] found small but consistent associations between physical inactivity and poorer mental health. Adolescents who are more physically active have a reduced chance of reporting depressive symptoms [12]. Other studies have also found that physical activity at age 15–16 years may influence some aspects of mental health three years later in boys, but not in girls [13].

Furthermore, young people who engage in sporting activities provide distinctive psychosocial benefits that protect them against suicidality [14]. As sports participation increases, the odds of suffering from depression and having suicidal ideation decreases [15]. In addition, the combination of high screen time and insufficient vigorous physical activity is associated with the highest prevalence of various psychological problems including depressive and anxiety symptoms [16].

Besides the effects of physical activity, dietary habits could also have a significant effect on mental distress in young people. For example, an association has been found between diet quality and depression where diet quality has the potential to modify mental health over the life course [17]. Similarly, breakfast quality could be an important component in the complex interaction between lifestyle factors and mental health in early adolescence [18]. O’neil et al. [19] also found that a significant cross-sectional relationship exist between unhealthy dietary patterns and poorer mental health. More specifically, disordered eating contributes to both suicide ideation and depressive symptoms [20]. Some studies have also shown that the choice to use vegetables in food may help individuals to separate themselves from disturbing thoughts [21] just as fruit and vegetable intake are significantly associated with lower odds of depression and distress [22]. Furthermore, consumption of fast food is associated with a higher risk of depression [23].

Although physical inactivity and poor dietary habits are known risk factors for the onset and/or perpetuation of mental distress in young people, parental involvement (parents showing concern in issues related to their children’s physical activity and dietary habits) may be a protective factor that could mitigate the deleterious effects of physical inactivity and poor dietary habits [24]. Traditionally, parental involvement require investments of time and money from parents such that those who are not able to provide these resources are deemed uninvolved [25]. However, this neglects the views and experiences of parents from low socio-economic backgrounds and minority populations regarding parental involvement [26]. Thus, it is only appropriate to assume that within the socio-economic and cultural context of Ghana, parents can demonstrate parental involvement through behaviours such as providing nurturance to their children, instilling cultural values, and talking with their children in order to understand their problems [27].

Accordingly, by showing affection in their children’s physical activity and dietary habits, parents powerfully shape children’s early experiences with physical activity and dietary behaviours which could in turn have a substantial effect on the mental health of adolescents. For example, parents could influence their children’s engagement in physical activity through their role modelling of physical activity and beliefs about their child’s competence [28] and through encouragement [29], which in turn could have a positive effect on mental health [18, 19, 20]. Furthermore, parental presence at evening meals is positively associated with adolescents’ higher consumption of fruits, vegetables, and dairy foods [30] which could in turn result in various mental health outcomes [21, 22, 23]. Again parental fruit and vegetable intake, knowledge of intake recommendations and skills have been found to have a positive association with children’s intakes [31]. As parents are responsible for regulating their children’s sugar-sweetened beverage consumption they are involved in interventions aimed at changing dietary habits in relation to consuming sugar-sweetened beverages [32].

From the foregoing, it is important to note that there is a paucity of literature examining the moderating or mediating role of parental involvement in a direct association between physical activity, dietary habits and mental distress among young people, especially in Ghana. As has been demonstrated, parental involvement enhances physical activity [29] and dietary habits [31] and both physical activity [12] and dietary habits [17] in turn have positive mental health consequences, suggesting the important role parental involvement plays in the development of children in terms of health and education [33, 34, 35]. Therefore, the aim of this study was to examine the role of parental involvement in the association between physical activity, dietary habits and mental health among Ghanaian youth.

## Materials and methods

### Data and participants

The study used secondary data from the 2012 Ghana Global School-Based Student Health Survey (GSHS). As required by GSHS guidelines, all due ethical protocol of the Ghana Education Service (GES) was followed. Informed consent was obtained from students who were 18 years old and above. For students who were less than 18 years old, informed consent was obtained from their parents/guardians after which these minors gave their assent before participating in the study. As stipulated by GSHS, participation in the study was voluntary, anonymous and confidential. The response rates was 74%. After the rationale of the analysis was outlined permission was obtained (ID 177964) before the 2012 GSHS data was downloaded from the GSHS website [36].

### Socio-demographic variables

Gender and age of the participants as captured in the survey, were the main demographic measures used. Socioeconomic Status (SES) was measured by using the variable, ‘During the past 30 days how often did you go hungry because there was not enough food in your home’. Response options ‘never’ to ‘rarely’ were recoded as 1—‘high socio-economic class’ and response options ‘sometimes’ to ‘always’ as 2—‘low socio-economic class’. Percentage of students who were overweight (greater than +1 SD from median for Body Mass Index (BMI) by age and sex) was used to assess whether or not youth were overweight. The responses were coded ‘No’ and ‘Yes’ [36].

### Sampling procedure

As per the GSHS, a two-stage cluster sampling design was used to obtain a representative sample of students. In the first stage, schools representing all 10 geographic regions of Ghana were selected. In stage two, schools were selected with a probability proportional to enrolment size and then classes within these schools were selected randomly such that all students in a selected class and school had an equal probability of participation. All student in these selected classes were eligible to participate.

### Measures

With deductions from previous studies socio-demographic variables, physical activity, dietary habits, and measures of mental distress were operationally extracted from the 2012 Ghana GSHS data as follows:

#### Physical activity

As a measure of physical activity participants responded to three questions that asked them about how often they engaged in physical activity: “During the past 7 days, on how many days were you physically active for a total of at least 60 minutes per day?” Response options were ‘No (0–4 days)’ and ‘Yes (5–7 days)’; “During the past 7 days on how many days did you walk or ride a bicycle to or from school?” Response options were ‘No (0 days)’ and ‘Yes (1–7 days)’; and “During this school year on how many days did you go to Physical Education (PE) class each week?”. Response options were ‘No (0–2 days)’ and ‘Yes (3 or more days)’ [37].

#### Dietary habits

To assess dietary habit the following three questions were asked: “During the past 30 days how many times per day did you usually eat fruit such as oranges, pineapple, watermelon, banana, guava, pear, sweet apple, mangoes, or pawpaw?”. Response options were ‘No (no—less than one time per day)’ and ‘Yes (2 or more times per days)’; “During the past 30 days how many times per day did you usually eat vegetables such as kontomire, garden eggs, lettuce, cabbage, okra, alefu, bira, ayoyo, or bean leaves?”. Response options were ‘No (no– 2 times per day)’ and ‘Yes (3 or more times per days)’; and “During the past 7 days on how many days did you eat food from a fast food restaurant such as vendors who sell pizza, hamburgers, fried chicken, fried rice, fried doughnuts, fried yams or potatoes, fried plantains, fried turkey (chofi), fried fish, or fried beef?”. Response options were ‘No (0–2 days)’ and ‘Yes (3–7 days)’ [38].

#### Mental distress

Mental distress was assessed with three indicators: Feeling lonely—“During the past 12 months how often have you felt lonely?”. Response options were ‘No (never—sometimes)’ and ‘Yes (most of the time, always)’; Feeling worried—“During the past 12 months how often have you been so worried about something that you could not sleep at night?”. Response options were ‘No (never—sometimes)’ and ‘Yes (most of the time, always)’; and Suicidal ideation–“During the past 12 months did you make a plan about how you would attempt suicide?”. Response options were ‘No’ and ‘Yes’ [39, 40].

#### Parental involvement

Parental involvement in their children’s physical activity and dietary habits was assessed by asking “During the past 30 days how often did your parents or guardians understand your problems and worries?” with two response options—‘No (never—sometimes)’ and ‘Yes (most of the time, always)’ [41]. The conceptualisation of this question is guided by the definition of parental involvement especially among low socio-economic status and minority populations [25]. Thus, the understanding of parental involvement within the socio-cultural context of Ghana would involve parents showing concern with respect to emotional supportiveness and warmth—where parents are seen as helpful, rewarding, nurturing, affectionate, and affiliative instead of being cold and emotionally depriving with specific reference to health behaviours [25].

### Statistical analysis

Statistical analysis was performed with IBM SPSS 22 software. Due to the complex nature of the data, complex samples option was applied in analysis, accounting for country-specific primary sampling unit, stratum, and sample weight. Multivariate logistic regression analysis with complex samples was used to examine associations between the dependent variable (mental distress), the independent variables (physical activity and eating habits), covariates (age, sex, BMI, and SES) and a moderator (parental involvement). In these analyses the socio-demographic characteristics of the participants were initially assessed against the three dimensions of mental distress. This was followed by an analysis that adjusted for socio-demographic characteristics while examining the effects of physical activity and dietary habits on mental distress. The final analysis also adjusted for socio-demographic characteristics while examining the interaction effect of parental involvement with physical activity and dietary habits on mental distress.

## Results

### Demographic characteristics of participants

The final analysis used a total of 1,984 high school students. The median age was 15 years with 53.7% (N = 1065) males. Only 7.9% were overweight and 13.3% lived in homes classified as low socio-economic class. Majority of the participants (53.7%) were 18 years or older and 44.6% of the total sample reported that their parents were involved in their physical activity and dietary habits. The prevalence of mental distress was 18.1%, 16.6% and 23% for loneliness, feeling worried and suicidal ideation respectively. Also, the frequency of dietary habits were: ate fruit 2+ times per day past 30 days (21.9%), ate vegetables 3+ times per day past 30 day (18.6%), ate fast food 3+ days past 7 day (24.8%) and that of physical activity were: active 60+ mins/day for 5+ of past 7 days (24.5%), walk/bike to/from school 0 of past 7 days (50.3%) and 3+ days PE each week (23.7%) respectively.

From Table 1 (before parental involvement was introduced into the analysis) it can be seen that males were significantly less likely to have suicidal ideation (OR = 0.67). Also, youth from homes classified as low socio-economic status were significantly more likely to report that they felt lonely (OR = 2.13), worried (OR = 2.12), and experienced suicidal ideation (OR = 1.98).

**Table 1:** Socio-demographic characteristics of participants (N = 1984).

	Category	N (%)	Lonely		Worried		Suicidal Ideation	
			OR (95% CI)	*t*	OR (95% CI)	*t*	OR (95% CI)	*t*
Age	11–17 years	915 (46.3)	1.21 (.56–1.70)	1.19	.89 (.68–1.17)	-.95	.88 (.72–1.08)	-1.34
	18 years +	1062 (53.7)	1		1		1	
	Missing	7						
Sex	Male	1065 (53.7)	.88 (.55–1.41)	-.60	.88 (.66–1.19)	-.90	**.67 (.46-.99)**	-2.25*
	Female	908 (46.3)	1		1		1	
	Missing	11						
Socio-economic Status	Low	261 (13.2)	**2.13 (1.38–3.28)**	3.78*	**2.12 (1.46–3.07)**	4.38*	**1.98 (1.27–3.09)**	3.35*
	High	1720 (86.8)	1		1		1	
	Missing	3						
Ate fruit 2+ times per day past 30 days	Yes	433 (21.9)	.98 (.69–1.39)	-.15	.72 (.50–1.03)	-1.98	1.61 (.97–2.65)	2.05
	No	1542 (78.1)	1		1		1	
	Missing	9						
Ate vegetables 3+ times per day past 30 days	Yes	368 (18.6)	.82 (.51–1.34)	-.87	1.28 (.87–1.88)	1.41	**.71 (.52-.98)**	-2.35*
	No	1608 (81.4)	1		1		1	
	Missing	8						
Ate fast food 3+ days past 7 day	Yes	491 (24.8)	1.16 (.80–1.68)	.88	1.16 (.80–1.67)	.87	1.24 (.93–1.65)	1.61
	No	1490 (75.2)	1		1		1	
	Missing	3						
Active 60+ mins/day for 5+ of past 7 days	Yes	481 (24.5)	.96 (.75–1.21)	-.41	**.68 (.49-.96)**	-2.46*	.89 (.58–1.35)	-.63
	No	1481 (75.5)	1		1		1	
	Missing	22						
Walk/Bike to/from school 0 of past 7 days	Yes	985 (50.3)	.97 (.76–1.23)	-.32	.93 (.61–1.40)	-.41	.88 (.67–1.16)	-1.02
	No	972 (49.7)	1		1		1	
	Missing	27						
3+ days PE each week	Yes	462 (23.7)	**1.23 (1.02–1.49)**	2.41*	.98 (.64–1.50)	-.09	1.02 (.86–1.21)	.27
	No	1484 (76.3)	1		1		1	
	Missing	38						
Parental involvement	Yes	867 (44.6)	.94 (.76–1.16)	-.66	.95 (.76–1.19)	-.50	**.60 (.43-.84)**	-3.30*
	No	1077 (55.4)	1		1		1	
	Missing	40						
Overweight	Yes	149 (7.6)	1.14 (.74–1.74)	.65	1.59 (.86–2.94)	1.65	**1.78 (1.13–2.80)**	2.77*
	No	1800 (92.4)	1		1		1	
	Missing	35						

*p < 0.05.

CI = Confidence Interval.

### Physical activity, dietary habits and feeling lonely

Table 1 shows that only one indicator of physical activity “attend 3+ PE classes each week” was significantly associated with loneliness (OR = 1.23). After adjusting for the effects of sociodemographic characteristics in a subsequent multivariate logistic regression analysis “attend 3+ PE classes each week” was no longer significantly associated with loneliness (Table 2). An interaction between parental involvement and physical activity and dietary habits after adjusting for socio-demographic characteristics showed that all the six indicators of physical activity and dietary habits were not significantly associated with loneliness (Table 3).

**Table 2:** Multivariate analysis of physical activity and dietary habit with mental distress.

Health behaviours		Lonely		Worried		Suicidal Ideation	
	Response	OR (95% CI)	*t*	OR (95% CI)	*t*	OR (95% CI)	*t*
Ate fruit 2+ times per day past 30 days	Yes	.95 (.67–1.35)	-.33	.71 (.49–1.01)	-2.10	**1.53 (.95–2.47)**	1.97*
	No	1		1		1	
Ate vegetables 3+ times per day past 30 days	Yes	.80 (.50–1.29)	-1.0	1.31 (.89–1.92)	1.51	**.73 (.54-.98)**	-2.34*
	No	1		1		1	
Ate fast food 3+ days past 7 day	Yes	1.13 (.78–1.65)	.72	1.14 (.79–1.65)	.78	1.25 (.95–1.66)	1.74
	No	1		1		1	
Active 60+ mins/day for 5+ of past 7 days	Yes	.94 (.73–1.21)	-.58	**.70 (.50-.98)**	-2.32*	.88 (.57–1.35)	-.67
	No	1		1		1	
Walk/Bike to/from school 0 of past 7 days	Yes	.96 (.75–1.22)	-.48	.91 (.62–1.35)	-.50	.90 (.68–1.18)	-.86
	No	1		1		1	
3+ days PE each week	Yes	1.19 (.98–1.44)	1.95	.96 (.62–1.49)	-.20	1.00 (.83–1.20)	-.06
	No	1		1		1	

* *p* < 0.05.

Analysis adjusted for age, sex, SES, and overweight. CI = Confidence Interval

**Table 3:** Multivariate analysis of the interaction between parental involvement and physical activity and dietary habits on mental distress.

Health behaviours		Lonely		Worried		Suicidal Ideation	
	Response	OR (95% CI)	*t*	OR (95% CI)	*t*	OR (95% CI)	*t*
Ate fruit 2+ times per day past 30 days X parental involvement	Yes x Yes	.83 (.48–1.45)	-.73	.56 (.30–1.05)	-2.01	1.10 (.49–2.47)	.25
	Yes x No	1		1		1	
Ate vegetables 3+ times per day past 30 days X parental involvement	Yes x Yes	.65 (.33–1.26)	-1.42	1.24 (.90–1.71)	1.48	.81 (.40–1.63)	-.67
	Yes x No	1		1		1	
Ate fast food 3+ days past 7 days X parental involvement	Yes x Yes	1.17 (.72–1.90)	.70	.94 (.63–1.41)	-.33	1.20 (.82–1.78)	1.03
	Yes x No	1		1		1	
Active 60+ mins/day for 5+ of past 7 days X parental involvement	Yes x Yes	.80 (.55–1.17)	-1.29	**.59 (.39-.90)**	-2.70*	.58 (.34–1.01)	-2.13
	Yes x No	1		1		1	
Walk/Bike to/from school 0 of past 7 days X parental involvement	Yes x Yes	1.25 (.82–1.91)	1.14	1.05 (.69–1.61)	.27	.77 (.51–1.19)	-1.31
	Yes x No	1		1		1	
3+ days PE each week X parental involvement	Yes x Yes	1.18 (.90–1.55)	1.32	1.26 (.69–2.31)	.83	.99 (.60–1.62)	-.06
	Yes x No	1		1		1	

* *p* < 0.05.

Analysis adjusted for age, sex, SES, and overweight. CI = Confidence Interval.

### Physical activity, dietary habits and feeling worried

Table 1 shows that only “active 60+ mins/day for 5+ of past 7 days” was significantly associated with feeling worried with youth who are “active 60+ mins/day for 5+ of past 7 days” less likely to feel worried (OR = 0.68). It can be observed from Table 2 that after adjusting for the effects of sociodemographic characteristics “active 60+ mins/day for 5+ of past 7 days” remained significant with youth who are “active 60+ mins/day for 5+ of past 7 days” being less likely to feel worried (OR = 0.70). When parental involvement was included ‘active 60+ mins/day for 5+ of past 7 days’ still had a significant association with feeling worried with youth “active 60+ mins/day for 5+ of past 7 days” being less likely to feel worried (OR = .59) (Table 3).

### Physical activity, dietary habits and suicidal ideation

Before the introduction of parental involvement into the analysis “ate vegetables 3+ times per day past 30 days” was the only health behaviour that was significantly associated with suicidal ideation (OR = 0.71) with youth who “ate vegetables 3+ times per day past 30 days” being less likely to experience suicidal ideation (Table 1). Table 2 shows that after controlling for the effects of socio-demographic characteristics “ate vegetables 3+ times per day past 30 days” remained significant (OR = 0.73). However, when parental involvement interacted “ate vegetables 3+ times per day past 30 days” was no longer significantly associated with suicidal ideation although there were increased odds of youth who “ate vegetables 3+ times per day past 30 days” being less likely to report suicidal ideation (OR = 0.81) (Table 3).

## Discussion

In this study we examined the role of parental involvement in the associations between physical activity, dietary habits and mental distress in Ghanaian youth. Prevalence in mental distress was relatively high which is consistent with previous studies that found a prevalence of 18.1%, 16.6% and 23% for loneliness, feeling worried and suicidal ideation respectively [42]. These findings are similar to other reported prevalence rates in Africa [4, 5] and the rest of the world [2, 43, 44]. These similarities in prevalence of mental distress in the present sample and in samples across Africa and the rest of the world confirms that mental health problems have become a common life experience of young people globally [45]. This is an indication that mental health problems such as feeling worried, lonely and having suicidal ideation are pervasive among the youth irrespective of their cultures, countries and poverty/wealth. Although a national intervention strategy aimed at improving the mental health of young people is recommended, this should be situated within the broader global context for various countries to benefit from each other’s strategies and interventions that have proven to be effective and successful. This finding among Ghanaian adolescents is timely and fits well within the context of the study by Kieling et al. [45] who reported that prevalence of mental health problems in children in low and middle income countries is similar to those in high income countries and recommended that future studies should address the imprecision of prevalence estimates to allow for improvement in services planning.

Results of this study on the association between socio-demographic characteristics and mental health problems showed that younger students were significantly more likely to feel lonely, worried, and have suicidal ideation compared to older students. In a 3-month longitudinal study which involved younger adolescents prevalence of anxiety disorders was high among this sample [46]. The significant age differences in mental health could be attributed to the series of challenges adolescents face in manoeuvring their transition period which are likely to predispose them to emotional and psychosocial struggles which could precipitate the high prevalence of common mental health problems. For example, younger students reported higher mental distress probably because young people encounter a series of age related challenges over the years as part of the process of human growth and development, but these challenges are more disruptive and challenging for younger adolescents [47].

Findings of this study also showed that youth from homes classified as low socio-economic status were significantly more likely to report that they felt lonely, worried, and experienced suicidal ideation. This is consistent with results of previous studies that children from socioeconomically disadvantaged families were approximately two to three times more likely to develop mental health problems than their peers from socioeconomically advantaged families [48]. According to Reiss [48] low household income and low parental education are the strongest predictors of mental health problems among children and adolescents and children from low SES families, especially with parents with a low education level, had limited access to structural resources such as mental health care. Children from higher socio-economic backgrounds have parents/guardians who are more educated with better occupations and incomes and are able to provide for the crucial needs of their children to make their children have less worries [49]. Thus, the association between socioeconomic inequality and mental health problems found among adults is also prevalent among adolescence.

Also, the results of this study showed that after adjusting for the effects of socio-demographic characteristics students who were physically inactive with poor dietary habits were more likely to report that they felt lonely, worried and experienced suicidal ideation. These findings are consistent with results of previous studies that found that physical activity has positive effects on mental health [11, 12, 15]. Sitting time (sedentary behaviour) can be high in the contexts of leisure time (e.g., screen time), school, and travel (i.e., car use) which has a direct relationship with weight gain and subsequent mental health problems [11]. Therefore, parents may need to be directly involved in participating in physical activity themselves for their children to model this behaviour [29]. Previous studies have also found that poor dietary habits have negative effects on mental health [17, 19]. This may be as a result of the fact that adolescents with internalizing disorders or symptoms eat more poorly as a form of self-medication [19]. Among the present sample it could be speculated that, psychosocially, students who engaged in physical activity were less likely to report mental distress because of the social interaction hypothesis that asserts that the social relationships and mutual support which those who engage in physical activity provide each other is responsible for a significant aspect of the effects of physical activity on their mental health [50]. This suggests that it is possible for students with fewer mental health problems to be more likely to seek out to their parents or guardians, socialise and then engage in various forms of physical activity, suggesting a reverse relationship between physical activity and mental health problems [51]. The inverse relationship between good dietary practices and mental distress could also be explained biologically in that dietary intake may have a direct effect on various biological systems and mechanisms that underpin depression, including oxidative processes, the functioning of the immune system, and levels of salient brain proteins [52]. Furthermore, green vegetables such as okra and fruits such as banana (in the present study) are good sources of magnesium and folate, both of which appear to play a role in depression [53].

Finally, after introducing parental involvement in the analysis, the six indicators of both physical activity and dietary habits were associated with loneliness, worry and suicidal ideation with decreased odds for adolescents to engage in some of these health impairing behaviours suggesting that parental involvement plays a crucial role in mitigating the negative effects of physical inactivity and poor dietary habits on loneliness, worry and suicidal ideation. This finding concurs with previous literature that emphasise the important role parental involvement plays in protecting young people against mental distress through the promotion of physical activity [29, 32] and good dietary habits [30, 31]. More parental support in the form of transport and enrolment leisure-time physical activity is required for organised physical activity among young people [29]. Similarly, the presence of at least one parent during the evening meal is associated with a lowered odds of poor consumption of fruits, vegetables, and dairy foods and a lowered odds of skipping breakfast among adolescents [30]. Finally, these findings are important because it serves as benchmark information and data on how parental involvement influence physical activity, dietary habits and mental health of Ghanaian youth. Studying the interrelationships among these variables simultaneously in a single study is one of the first empirical studies to be conducted on this topic in Ghana.

### Limitations of the study

Limitations of this study include the fact that as the GSHS used a cross-sectional data this study could not infer causality between parental involvement, health behaviours and mental distress. A longitudinal study or data would be the best way to establish and infer causal relationships among these variables. Also, the measurement of mental health—feeling worried, lonely and having suicidal ideation were assessed by single item questions confined to the existing GSHS dataset making it quite narrow in the assessment of mental ill-health. Although not sufficient for diagnostic purposes, these questions could represent and embody non-clinical mental distress among adolescents in Ghana today. Finally, the use of a single item as a measure of parental involvement may not have given a holistic assessment of this construct in relation to youth physical activity, dietary habits and mental distress as it concentrated largely on parents understating the problems and worries of their children in relation to physical activity and dietary habits. Parents understanding their children’s problems and worries in relation to physical activity and dietary habits may not necessarily mean that they are involved in these health behaviours. Therefore, interpretation of the results of this study should be done with caution. These notwithstanding, to the best of our knowledge, this is the first cross-sectional study that has simultaneously examined how parental involvement could mitigate the effects of physical activity and dietary habits on mental distress in Ghanaian youth.

### Conclusion

This study examined the role of parental involvement in the physical activity and dietary habits and how this impacted on the mental health of adolescents. Physical inactivity and poor dietary habits have a substantial effect on mental health of school going youth in Ghana. However, parental involvement mitigated the deleterious effects of negative lifestyle habits on mental distress. Therefore, there is the need to introduce and strengthen public health interventions targeted at promoting healthy lifestyles, especially physical activity and dietary habits among Ghanaian youth. It is recommended that the Ministry of Health and the Ministry of Education should liaise to ensure that physical education lessons are taken serious by various schools to ensure that students actively participate in these lessons. Also, food sellers in schools should be mandated to include fresh fruits and vegetables alongside the food they sell to encourage students to buy these fruits and vegetables. Accordingly, schools should also serve fresh fruits and vegetables in the dining halls for students who are in the boarding houses. Furthermore, schools should create conducive avenues where students can engage in sporting activities. The same way, communities, through District Assemblies, should construct more recreational grounds and playing fields so that young people will get motivated to engage in some physical activities.

It is also important that parents and guardians are encouraged and equipped with effective parenting skills to get involved in their children’s physical activity and dietary behaviour. These suggestions or advice could be given to parents by teachers through their regular parent-teacher association meetings or through public health advertisements. The national commission on civic education could also embark on nation-wide parental education campaigns where parents would be made aware of the importance of getting involved in their children’s physical activity and dietary habits as these have profound consequences on their children’s mental health.
